# Role of Th17 Cells in Skin Inflammation of Allergic Contact Dermatits

**DOI:** 10.1155/2013/261037

**Published:** 2013-08-18

**Authors:** Matthias Peiser

**Affiliations:** German Federal Institute for Risk Assessment (BfR), Department of Chemicals Safety, Max-Dohrn-Strasse 8-10, 10589 Berlin, Germany

## Abstract

Extending the classical concept considering an imbalance exclusively of T helper(h) 1 and Th2 cells on the bottom of many inflammatory diseases, Th17 cells were recently described. Today, there is sufficient experimental evidence to classify psoriasis and allergic contact dermatitis (ACD) amongst other inflammatory skin disorders as IL-17 associated diseases. In several human studies, T-cell-clones could be isolated from eczema biopsies, and high IL-17 levels were observed after challenge with allergen. In the last years, the phenotype of these IL-17 releasing T cells was in the focus of discussion. It has been suggested that Th17 could be identified by expression of retinoic acid receptor-related orphan receptor (ROR)C (humans) or ROR**γ**t (mice) and IL-17, accompanied by the absence of IFN-**γ** and IL-22. In cells from skin biopsies, contact allergens elevate IL-17A, IL-23, and RORC within the subset of Th cells. The indications for a participation of Th17 in the development of ACD are supported by data from IL-17 deficient mice with reduced contact hypersensitivity (CHS) reactions that could be restored after transplantation of wild type CD4^+^ T cells. In addition to Th17 cells, subpopulations of CD8^+^ T cells and regulatory T cells are further sources of IL-17 that play important roles in ACD as well. Finally, the results from Th17 cell research allow today identification of different skin diseases by a specific profile of signature cytokines from Th cells that can be used as a future diagnostic tool.

## 1. Introduction

“…The immunizing activity of conjugated antigens comes into play, this concept affording a plausible explanation for the immunological effects of simple substances”. Even today not all physicians, toxicologists, and health care professionals are aware of what Landsteiner and Chase 1937 supposed [1], which is that inflammatory events caused by the immune system are at the bottom of the disease allergic contact dermatitis (ACD). In his “Anaphylaxis Experiments,” he reported skin sensitization in guinea pigs after treatment with picryl chloride and 2,4-dinitrochlorobenzene (DNCB). More than 70 years later, we identified different subpopulations of lymphocytes to participate in human ACD and mouse hypersensitivity reactions. However, on the distinct underlying mechanisms, how IgE (B cell) and type IV (T cell) mediated reactions could be linked, we can only speculate. Obviously, the interplay of different skin and immune cells, cytokines, chemokines, and further mediators in ACD is more complex than a simple T helper(h)1/Th2 imbalance would explain. In respiratory and dermal allergic reactions, higher tissue levels of IL-17 were observed, and different cells were proposed to be the major cytokine source. In this review, we summarize recent findings on the model of the innate and adaptive immune mechanisms in contact allergy and further focus on the role assumed for the newly described Th17 cells. 

## 2. Contact Dermatitis: Inflammation of the Skin by Complex Immune Mechanisms 

 ACD is an inflammatory skin disease in humans that appears by a rash on the skin after exposure to xenobiotics or haptens. In complex with protein, a hapten generates a full allergen, and sensitization (first phase) is followed by the elicitation phase after re-exposure with the same allergen. ACD is typically accompanied by skin lesions, the allergic contact eczema, that is caused by delayed type (type IV) immune reactions. Studies on ACD can be conducted by the experimental model of contact hypersensitivity (CHS) in mice. An irritative contact dermatitis and irritative eczema were originally classified as nonimmunological cutaneous inflammatory responses [2]. As inflammation in the complete absence of immune cells is difficult to imagine, the allergic and irritative forms are now distinguished from each other to indicate if hapten-specific T cells of the adaptive immune system are involved or not [3]. Different from atopic diseases, IgE is typically not enhanced in ACD. The first step of the development of an ACD is one of the most enigmatic and should not be discussed here in detail: chemicals of low molecular weight (<0.5 kDa) or metal ions enter the cornified upper layers of the epidermal skin by penetration [4]. A direct access to the deeper skin sections of the dermis could be facilitated by mechanical, sunburn, irritation, or infection-induced rupture of the epidermal barrier. Prohaptens are suspected to be activated by the host metabolism; prehaptens are activated externally by autoxidation [5]. The result from covalent binding to one or more carrier-proteins is the construction of an antigenic hapten-protein complex; the result from noncovalent interaction of metal salts with amino acids is chelation complexes [6]. 

**Figure 1:**
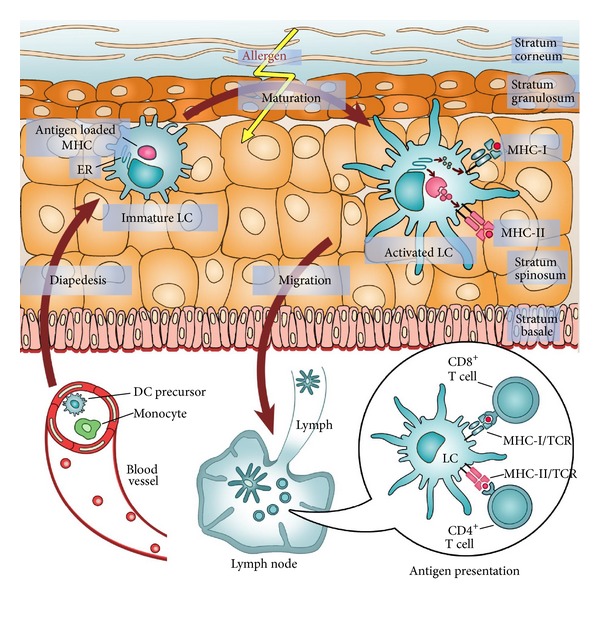
Skin exposure to allergens induces maturation of LCs and migration to regional lymph nodes. After disrupture of the epidermal barrier, haptens such chemicals gain access to the deeper compartment of the skin. Even in steady state, sentinels of the immune system, LCs, move their protrusions between the keratinocytes to the tip of the inner cornified cell layer to sample antigens. If an allergen is detected in the context of danger, the antigen-loaded LC leaves the epidermis and migrates to the lymph node where cytotoxic T cells and T helper cells are stimulated to proliferate and acquire a specific phenotype. After skin inflammation, LCs are recruited from blood monocytes and repopulate the epidermis. DC, dendritic cell; LC, Langerhans cell; MHC, major histocompatibility complex; TCR, T cell receptor.

**Figure 2:**
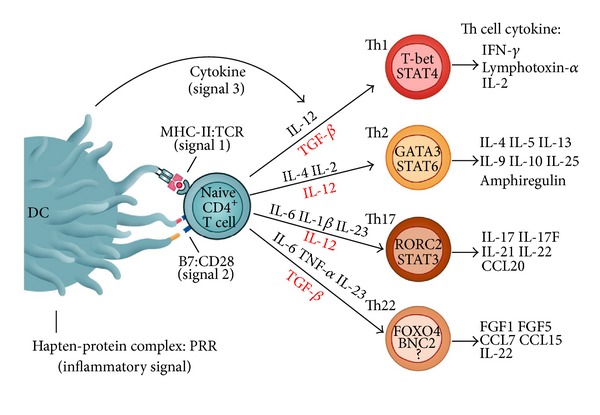
Polarization of different Th cell subpopulations by one inflammatory signal and three DC derived signals. Skin resident LCs and dermal DCs recognize a complex of skin penetrating haptens and proteins via PRRs. Subsequently, these cells mature and display antigenic epitopes to TCR bearing T cells in the lymph node. The allergen is presented by MHC molecules (signal 1) and costimulatory molecules deliver a signal 2. The profile of differentiating cytokines and growth factors released by activated DCs is crucial for the development of individual Th cells. These are characterized by expression of transcription factors such as T-bet, GATA3, RORC (human counterpart of murine ROR*γ*t), FOXO4, and release of signature cytokines such as IFN-*γ* for Th1, IL-4 for Th2, IL-17 for Th17, and IL-22 for Th22. PRR, pattern recognition receptor; Th, T helper cell.

Undoubtedly, it is a dendritic cell (DC) that finally decides if a detected molecule will be regarded as harmless if derived from some commensal bacteria and self-peptides or as foreign if derived from pathogenic microorganism and from “altered-self” molecules. The consequence is the initiation of tolerance or immunogenicity. The question remains what subtype exactly of DC executes the tasks of antigen sampling, processing, presentation, and lymphocyte activation? It was the firstly described DC, the Langerhans cell (LC), that for a long time and for some experts still is the skin-resident cell type that primes at least naïve lymphocytes and thus connects the innate with the adaptive immune system [7, 8]. According to the original concept, DCs including dermal DCs (DDCs) and LCs could prime naïve T cells in skin draining lymph nodes [9]. These professional antigen presenting cells could show antigenic peptides or haptenized proteins exhibiting neoepitopes in the binding groove of major histocompatibility complex molecules (MHC). In the lymph node, allergen specific T cells form an immunological synapse with DCs and recognize “their” epitopes presented on MHC molecules by the cognate T cell receptor (TCR, Figure 1) [10]. Therein, signals are transmitted between APC and T cells in a bidirectional way by interaction of MHC:TCR (signal 1), adhesion molecules CD48 and CD59:CD2, ICAM1:LFA1, and costimulatory molecules such as CD86/CD80:CD28 (signal 2, Figure 2). In contrast, APCs restrict, diminish, or shut off specific T-cell responses via negative signals, if members of the B7:CD28 family induce tolerance [11]. In ACD and hypersensitivity models, a crucial inhibitory function was reported for B7 molecules and their ligands CTL-A4 [12–14], ICOSL:ICOS [15, 16], and PD-L1/-L2:PD-1 [17–19]. However, in response to a complete interaction of activating molecules, allergen-stimulated APCs release cytokines such as IL-12, IL-6, and IL-23 that could drive T-cell polarization. Dependent on the cytokine milieu, Th1, Th2, Th17, and cytotoxic type 1 T-cells (Tc1) differentiate. After expression of homing receptors, they could migrate into the skin where the activating DDCs or LCs originate from [20]. 

In the last years, LC's crucial role in initiation of a skin immune response after antigen uptake, migration into draining lymph nodes, and antigen presentation was, however, questioned [21]. Against expectation, in LC-deficient mice reduced and even enhanced but no disabled hypersensitivity reactions were observed, and thus it was assumed that LCs might be dispensable for induction of ACD in animal models [22, 23]. Instead, DDCs were shown to participate in hypersensitivity reactions [24]. After allergen-challenge, DDCs enter auricular lymph nodes prior to LCs [25]. Moreover, they activate T cell proliferation in PAF receptor-deficient mice after stimulation with 2,4-dinitrofluorobenzene (DNFB) [26]. On the other hand, it was recently demonstrated in LC ablation models that LCs could initiate epicutaneous sensitization by OVA-specific IgE and thereby induce a Th2 response [27]. However, in Langerin-Cre MyD88(fl) mice, where LCs fail to recognize most Toll-like receptor (TLR) ligands including allergens, no effects on hypersensitivity reactions were observed [28]. Finally, the precise role of LCs in the molecular mechanisms of ACD is still under debate, but there are several hints indicating that LCs may accomplish an extended function in ACD by mediating tolerance [29]. Our group could recently demonstrate a regulatory potential of LCs on cytokine release of Th cells through expression of regulatory B7 proteins including programmed death-ligand 1 (PD-L1) [19]. But an intrinsic control function of LCs to avoid further tissue damage in eczema lesions does not necessarily exclude an initiator function under different, more inflammatory conditions. In addition, new proofs were brought for the assumption that LCs are even in steady state highly mobile cells that do not passively wait on the bottom of the basal lamina for skin penetrating haptens. Instead, they actively crawl for potential allergens/antigens by extending their cellular protrusions between the keratinocytes in all epidermal layers until the stratum corneum [30]. In addition to DCs, other potentially antigen presenting cells (APCs) were proposed to be involved in ACD. In keratinocytes, contact sensitizers were shown to induce NALP3-inflammasome triggered release of IL-1*β* and IL-18, cytokines which presence is mandatory for DC maturation and migration [31]. Beneath this support of effective antigen presentation by haptens operating as danger signals, activated keratinocytes rather restrict contact allergy, because expression of high levels for MHC-I and -II molecules together with weak CD80 and IL-10 release preferentially leads to T cell anergy [32, 33]. Overall, the nature of the APC has together with the stimuli profile great impact on the quality of the adaptive immune response including Th17 differentiation. With respect to human, cutaneous resident DCs, for example, LCs isolated from normal epidermis were found to polarize Th22 more pronounced than DDCs derived from human dermis [34], IL-17 was lacking in the absence of stimulation. On the other hand, primary human LCs were shown to elicit greater IL-17 response in total CD3^+^ cells than DDCs, in both conditions unstimulated and after TLR stimulation [35]. 

The different capacity of distinct subsets of DCs and inducible APCs may rely on their individual expression of pathogen recognition receptors (PRRs) such as TLRs, complement receptors, and cytosolic proteins of the nucleotide-binding oligomerization domain-containing proteins (NOD1 and NOD2). In addition to antigen recognition process, TLRs facilitate APCs to sense evolutionary conserved structures, pathogen-associated molecular pattern (PAMP) that originate from pathogenic virus, yeast, protozoa, or bacteria [36, 37]. Though expressed in various cell types, DCs and LCs demonstrate the most complete arsenal of intracellular and surface TLRs [38, 39]. In the presence of microbial stimuli such as LPS or peptidoglycan, stimulation with toxins or irritants or DAMPs (damage-associated molecular patterns), TLR bearing DCs release specific cytokines (signal 3) that exhibit the capacity to differentiate Th cell subsets [4, 40]. In the skin, DAMPS such as reactive oxygen species (ROS), ATP and low-molecular-weight hyaluronic acid could even be generated by contact allergens. Otherwise, the metal ions Ni^2+^ and Co^2+^ were reported to stimulate innate immunity directly via TLR4 and its coreceptor MD2. Thus, a recent model suggested Ni^2+^ and TLR4 as an “inflammatory signal” for induction of ACD by allergen: PRR interactions [41, 42]. An inflammatory signal may also be contributed by sensitizing haptens that target the inflammasome. Key elements of this multienzyme complex are pyrin-like protein NLRP3, the adaptor ASC, and IL-1*β*/IL-18 precursor cleaving caspase 1. Using specific knockout models addressing these proteins, several haptens were proven to affect inflammasome associated IL-1*β* and IL-18 release [31, 43, 44]. In addition, haptens can induce release of ATP in epidermal cells and thereby activate the NLRP3 inflammasome of neighbouring cells expressing purinoceptor P2X7 [45]. It was suspected that both an allergen-specific MHC:TCR signal and an inflammatory signal are mandatory for complete T-cell activation (Figure 2). Consequently, these PRR and inflammasome activating haptens could be regarded as “false” danger signals that enter, in the absence of danger, the molecular mechanisms of “true” danger signals [46] such as bacterial cell wall compounds.

## 3. Th17: New Players in ACD

### 3.1. Generation of Th17: Signals Required, Relation to Other Th Cells

Polarization of Th cells is a stochastically regulated process in which the nature and concentration of the antigen or allergen, the exposure and environmental factors, and the strength and duration of TCR stimulation are critical factors. In addition, cytokines, chemokines, and growth factors are released by APCs within the immune synapse after antigen recognition and migration to the lymph node. If we apply the current concepts of antigen presentation and effective T cell priming via MHC/antigen recognition by TCR (signal 1), costimulation via B7:CD28 (signal 2), a distinct cytokine pattern (signal 3) [47], and an inflammatory signal [41], we could summarize the following: an inflammatory signal contributed by the skin penetrating allergen is mandatory to activate and mature LCs or DCs, which subsequently transmit the activation by three further signals to naïve T cells within the lymph node (Figure 2). Thus, Th17 cells and other T cell subpopulations were induced even in ACD by a complex signalling network. Th1 cells arise under the influence of IL-12p70; for polarization of Th2 the presence of external IL-4 is required [47]. Recently, the Th1/Th2 paradigm [48] was extended by the description of Th17 and Th22 cells that were characterized by expression of the exclusive release of cytokines and chemokines, receptors and transcription factors. IL-17 is regarded as a signature cytokine for Th17 cells and is involved in inflammatory responses as observed in bacterial and fungal defense mechanisms, development of autoimmunity, tumors, and allergic reactions such as CHS, Th2 delayed type hypersensitivity and asthma [49]. In ACD, the activity of cytotoxic T cells becomes evident in the appearance of skin lesions, but subpopulations of Th cells also contribute by the release of a specific cytokine pattern. 

#### 3.1.1. Murine Th17

The function of Th17 differentiating factors and Th17 associated diseases were investigated in specific mouse models for infection and autoimmunity. A crucial role for Th17 in defense of extra- and intracellular bacteria and fungi was reported [50–52]. One of the first indications for involvement of Th17 in autoimmune diseases came from cytokine replacement studies identifying IL-23, but not IL-12, as a key activator of macrophages in the pathogenesis of myelin oligodendrocyte glycoprotein (MOG) induced experimental autoimmune encephalomyelitis [53]. In rheumatoid arthritis, Th17 cells were shown by specific knockout models to promote osteoclastogenesis and bone resorption [54]. In murine models of intestinal diseases, a proinflammatory function of IL-23 and IL-17 was observed [55, 56]. In general, Th17 cells were generated from their precursors by the local presence of a specific setting of cytokines released by different neighbouring cell types such as DCs or monocytes. In studies using the murine system, it was demonstrated that IL-1 induces differentiation of Th17 cells IL-6 inhibits the T_reg_ pathway and thereby allows TGF-*β* to act as a Th17 driving factor. Under the influence of IL-21 Th17 could amplify, at the presence of IL-23 and its receptor they remain their phenotype [57]. Th17 themselves show the capacity to release IL-17A, IL-17F, IL-21, IL-22, and TGF-*β*. Murine Th17 are further characterized by expression of retinoic acid receptor-related orphan receptor (ROR)*γ*t [58]. In a new approach of transcriptional profiling at high temporal resolution, the chromatin regulator Mina, the TNF receptor Fas, Pou2af1 (OBF1), and Tsc22d3 were impressively proposed as new, specific factors of murine Th17 cells [59].

#### 3.1.2. Human Th17

Interestingly, species differences exist, for example, for the IL-23 presence, chemokine receptor expression patterns, development of the cell phenotype before reaching the homing tissues, and in the inhibitory capacity of Th1/Th2 factors on the Th17 pathway. In parallel to murine Th17 expressing ROR*γ*t, generation of human Th17 cells is accompanied by expression of nuclear receptor RORC2 [60]. RORC2 transduced human CD4^+^ T cells released IL-17A, IL-22, IL-6, and TNF-*α* [61], but RORC2 is challenged for being a specific, exclusive transcription factor for human Th17 by the fact that T_regs_ were shown to exhibit RORC2 as well [62]. In addition, human IL-17 producing cells and not Th1 or Th2 are derived from CD4^+^ T cell progenitors that are characterized by expression of CD161 [63]. They also exhibit a specific chemokine receptor profile demonstrated in subpopulations with a CCR4^+^/CCR6^+^ and a CCR2^+^/CCR5^−^ phenotype [64, 65]. More recently, NO-specific synthase 2 (iNOS) expression and signaling (cGMP-dependent protein kinase pathway) were discovered to be mandatory for induction and stability of human Th17 cells [66]. A phenylalanine oxidase (IL4I1) was also enhanced on Th17 cells [67]. Preferentially in the human system, Th cells with a mixed phenotype were described. A subset of Th1/Th17 cells was reported, which expresses both master regulators of transcription, T-bet and RORC. Finally, it was hypothesized that Th17 cells coreleasing IL-22 were Th17 cells in an immature stage [68]. IL-17 could also be induced by bacterial cell wall compounds, enterotoxins (superantigens), and cytolysins. In our group, bacterial, TLR2 activating stimuli were shown to induce Th17 generating cytokines in LCs and thereby a Th1/Th17 phenotype in CD4^+^ T cells [69]. In cocultures of immature DCs and mite allergen-specific T-cell clones, a strong release of both IL-17 and IFN-*γ* was observed after exposure to staphylococcal enterotoxin B [70]. In parallel, supernatant of alpha-toxin (from *Staphylococcus aureus*) stimulated monocytes induced IL-17 secretion in allogeneic CD4^+^ T cells [71] (Table 1).

### 3.2. Th17 in ACD

Indications for involvement of Th17 in human skin allergy were reported for the first time by detection of IL-17 mRNA in skin lesions from nickel allergic patients and in skin-derived nickel specific T cells [77]. After activation with PMA and ionomycin, exclusively CD4^+^ T cells released protein for IL-17 in the supernatant. A coexpression of IL-17 with IFN-*γ* was also observed, but the cell source was not detected. Evidence for a function of CD4^+^ T cells in CHS was provided by studies with IL-17 deficient mice that demonstrated strongly reduced ear swelling response to contact allergens [72]. Further discrimination of the total CD3^+^ T-cell population showed that it was the CD4^+^ T cells not the CD8^+^ T cells that decreased cell division in IL-17^−/−^ mice. In cell transfer experiments where CD4^+^ T cells originating from wild-type mice were transplanted in IL-17^−/−^ mice, ear swelling response in reconstituted mice was recovered comparable to wild-type mice after application of TNCB. Further hints for the existence of Th17 were given after observations in biopsies of patients with ACD. In contrast to vehicle controls, IL-17 and IL-22 single positive cells and CD4^+^CCR6^+^ T cells were observed in immunohistochemical analyses [73]. If inflamed skin tissue infiltrating IL-17^+^/IL-22^+^ double positive cells were present in situ was not reported. Interestingly, proliferating CD45RO^+^ Th cells releasing IL-17 were detected only after stimulation of autologous T cells with Ni^2+^ pulsed DCs that were generated from peripheral blood of patients with an allergy to nickel. This study clearly demonstrated the presence of memory Th17 in the blood of individuals having an allergy to nickel and their capacity to release IL-17 after rechallenge. If the skin derived (CCR6^+^) Th17 cells were the cells that secrete IL-17 and if the Th cells were coexpressing IL-22 could not be concluded because costaining was not performed. In a further study with five patients with psoriasis and ACD, cytokine levels were detected in supernatants of CD3/CD28-activated or NiSO_4_-reactive T cells that were isolated from eczematous and psoriatic lesions [78]. High levels of IL-17 and IL-22 were detected in both types of skin lesions after CD3/CD28 stimulation and were obviously different from small amounts detected in T cells from patients with atopic eczema. Interestingly, significantly higher levels of IL-17 and IL-22 in NiSO_4_-reactive T cells were observed in allergic eczema tissue than in psoriasis lesions. However, the peak of IL-17 was at about 100 pg/mL per million cells which is relatively low. Because costaining of IL-17 and IL-22 was not performed, it was not possible to discriminate Th17 (RORC^+^IL-17^+^) and Th22 (IL-17^+^IFN-*γ* ^+^IL-22^+^) cells [74]. The participation of Th17 at the total amount of IL-17 secreting T-cells was determined by analyses of subpopulations in human skin biopsies [75]. In T-cell lines, costaining for intracellular IL-17 and CD4 and CD8 revealed more than 90% Th cells in the population of IL-17^+^cells. If nickel was used for stimulation, T cells were infiltrating rather with a Th1/IL-17, Th0/IL-17, and only few with a Th2/IL-17 phenotype. Pure hapten-specific Th17 cells were not found under the T cell clones isolated. If cytokine coexpression was not due to PMA with ionomycin treatment, the appearance of IL-17^+^IFN-*γ* ^−^ cells as possible sources for IL-17 in nickel allergy remains obscure. Interestingly, the percentage of Th17 in the total IL-17^+^cells in ACD T cell lines varied by the usage of different allergens such as thiuram and nickel. A study analyzing primary human T cells from patients with psoriasis, atopic eczema, and ACD suggests that ACD can be distinguished from the other skin disorders by specific cytokine profile including IL-17 [76]. In direct comparison, IL-17 expression was quantified in skin biopsy cells from patients with psoriasis (17%), ACD (13%), and atopic eczema (9%). Of note, no exclusive expression of the Th lineage-associated cytokines IL-4, IL-17, IL-22, and IFN-*γ* was observed in the skin disorders examined, but characteristic coexpression pattern was described. Moreover, only a minority of IL-17^+^ and IL-22^+^ cells was single positive entities. In a study using skin biopsies of nine patients allergic to different contact allergens, upregulation of IL-17A, IL-17F, IL-23, and RORC was detected [79]. A closer look revealed that about 30% and 20% of the RORC^+^ cells were CD4^+^ and CD8^+^ cells, respectively. In a mouse study, the suppression of Th17 cells by prostaglandin E2 receptor (EP4) antagonist in CHS was demonstrated [80]. In controls without antagonist, 2,4-dinitrobenzenesulfonic acid sodium salt (DNBS) induced the release of IL-17. However, even if the IL-23-mediated Th17 cell amplification in vitro was shown for isolated normal CD4^+^ T cells, the total lymph node cells in the CHS experiments were not specified for T-cell populations. Using the official OECD assay for sensitization testing of chemicals, the local lymph node assay, DNFB was demonstrated to increase transcripts of IL-17 in cells isolated from excised, murine ear, and thymus [81]. 

In case of a crucial role of Th17 in ACD as effector cells and because of their fatal effect on target cells such as recruitment of neutrophils [82], activation of fibrocytes [83], and macrophages [84], Th17 themselves must be controlled by feedback mechanisms, restricting excessive tissue damage. Such a regulatory function was recently described by our group for coinhibitory molecules on primary LCs and their interaction with cognate receptors on cocultured CD4^+^ T cells [19]. In detail, it was a subpopulation of CCR6^+^/CCR4^+^ cells that released increased amounts of IL-17 after anti-PD-L1 was administrated to nickel triggered LCs. In addition, we found high expression of PD-L1 in skin biopsies of ACD patients after nickel challenge. Thus, we suspected that the expression and binding of PD-L1 to PD-1 (an inhibitory signal 2) mediates a control of LCs on IL-17 release from Th cells and concluded that this PD-L1-PD-1 signalling feedback loop may represent a mechanism to avoid excessive cytokine release during ACD related progress of eczema. Further, different molecules were shown as well to restrict IL-17 in contact allergy such as in experiments with OVA-induced cutaneous delayed-type hypersensitivity models. In annexin A1 deficient mice elevated release of ROR*γ*t and IL-17A by CD4^+^ T cells was detected [85]. In a further CHS model with disrupted TGF-*β* signalling, oxazolone (OXA) induced in Smad3-deficient mice significant increase of IL-17 mRNA [86]. Total cells were analyzed in skin biopsies from OXA-exposed skin sites and ear draining lymph nodes. CD69 was demonstrated as well to regulate responses of OXA-specific T cells. Irrespectively if used CD69^−/−^ or wild type mice, neutralizing anti-IL-17 strongly diminished CHS associated ear swelling [87]. Interestingly, in knockout mice, an increase of IL-17 from lymph node cells correlated with a higher percentage of positive cells, more prominent for CD4^+^ than for CD8^+^ cells. Finally, specific peptidoglycan recognition proteins were also shown to limit the Th17 response in an OXA-CHS model [88]. 

### 3.3. Further T Cell Populations as Sources of, IL-17 in ACD

Within the population of T cells, IL-17 was also observed in CD8^+^ T cells. Remarkably, in a DNFB induced CHS model, ear swelling was more reduced in CD8 depleted than in CD4 depleted mice, indicating a relevance of CD8 in ACD [89]. Isolated in culture with IL-23 and hapten-labelled DCs, these primed CD8^+^ T cells released higher amounts IL-17 than CD4^+^ T cells. A CD8^+^IL-17^+^IFN-*γ* ^−^ population was identified and discriminated from Tc1. Nevertheless, both cytokines are required for the elicitation reaction in CfrHS [90]. In this study, analyses of the phenotype revealed that in lymph node cells from DNFB-sensitized mice, CD4^+^ and CD8^+^ T cells comprised IL-17 single positive cells and a small amount coexpressing IFN-*γ*. It was further demonstrated in knockout mice that for expression of IL-17 and IFN-*γ* by CD8^+^ T cells in the hapten-challenge site, the presence of adhesion molecule ICAM-1 and IL-1 receptor was required [91, 92]. Thus, endothelial cell presentation of hapten and intact IL-1 receptor signalling might be an exclusive prerequisite for CD8^+^ T cell mediated IL-17 secretion. The presence of IL-17 could enhance CD54-dependent adhesiveness of Th1 lymphocytes to a monolayer of human autologous keratinocytes and induces killing in an antigen independent way. These findings suggest non-specific cytotoxicity occurring in ACD as well [75]. Decreased and increased ear swelling was observed after anti-CD8 and anti-CD4 treatment, respectively, and together with a study using MHC restricted T cells, a critical role of CD8^+^ T cells in DNFB-CHS was deduced [93, 94]. Vice versa, these observations in mice do not exclude specific functions of IL-17 releasing CD8^+^ and CD4^+^ T cells in the successive phases of sensitization and elicitation during ACD. Furthermore, with the proposal of a new regulatory T cell subtype (IL-17^+^ICOS^+^T_regs_) [93], a minimum of three T cell subsets, similar in the capacity to release IL-17, but all with specific roles in CHS or ACD, are described. In the human system, however, in supernatants of blood derived T cell clones with a CD8 T cell phenotype from patients with ACD to nickel, neither IL-17 release nor transcripts in the cells were detected [77]. After all, a discussion and further studies on the individual phenotype rather than on the amounts of IL-17 in distinct T cell subtypes in an individual condition would be helpful to improve efforts in substantiating the function of Th17 and IL-17^+^CD8^+^ T cells. For the sake of completeness concerning cellular sources of IL-17, innate IL-17 producing T cells and neutrophils were reported. All these populations, *γδ* T cells, invariant natural killer T (iNKT) cells, lymphoid-tissue inducer (LTi)-like cells, natural killer (NK) cells, and neutrophils have been identified as important IL-17 sources [95]. Anyway, with exception of neutrophils in eczema [96] a pivotal role of IL-17 derived from these specific populations in skin diseases including ACD has to be elucidated.

## 4. Concluding Remarks

Up to now there is fundamental data provided by several studies on CHS in genetically modified mice and on cells isolated from human eczema lesions supporting a crucial role of Th17 in ACD. From the current view, Th17 cells are characterized by expression of CD4, RORC, and IL-17 [74]. For discrimination from Th22, a costaining with IFN-*γ* and IL-22 was suggested, and this should be regarded as mandatory to identify Th17. Blocking studies with antibodies against the p40 subunit of IL-23, a cytokine that interferes in the expansion and stabilization, not in the induction phase of Th17 development, indicate that complex and probably redundantly regulated mechanisms must be assumed in therapeutic treatment of ACD [97]. In parallel, the absence of p40 dependent Th1 in ACD could not be concluded as well. Several new studies with cells from human eczema lesions delivered experimental evidence for the presence of Th17 in ACD and isolated Th17 cells lines released significant amounts of IL-17 in response to chemical haptens. Collectively, in addition to psoriasis and pathogen related skin disorders, there are significant experimental proofs pointing to an involvement of Th17 in ACD. 

## Figures and Tables

**Table 1:** Summary of studies on human Th17 cells. T-cell lines were derived from skin biopsies by limiting dilution and further specified for CD4 or not. Skin cells were analyzed without specification of the phenotype. In some studies, coexpression of IL-17 with cytokines specific for Th1, Th2, or Th22 was analyzed by flow cytometry.

T-cell subset	Patients	Hapten	Cytokines detected	Coexpression analyzed	Reference
CD4^+^ T cells	ACD	NiSO_4_	IL-17, IFN-*γ*, TNF-*α*	No	[71]
CD4^+^ CD45^+^ T cells	ACD	NiCl_2_	IL-17, IFN-*γ*	No	[72]
T cells	Psoriasis, ACD	NiSO_4_	IL-17, IFN-*γ*, IL-22, IL-4	No	[73]
T cells	ACD	Fragrances	IL-17, IFN-*γ*, IL-22, IL-4	Yes	[74]
T cells	ACD	NiSO_4_	IL-17, IFN-*γ*, IL-4	Yes	[74]
T cells	ACD	Nickel	IL-17, IFN-*γ*, IL-22, IL-4	Yes	[75]
Skin cells	ACD	Nickel	IL-17, IFN-*γ*	No	[76]

## Abbreviations

ACD: Allergic contact dermatitis
APC: Antigen presenting cell
CHS: Contact hypersensitivity
DAMP: Damage-associated molecular pattern molecule
DC: Dendritic cell
LC: Langerhans cell
OVA: Ovalbumin
PAMP: Pathogen-associated molecular pattern
PRR: Pattern recognition receptor
Tc: Cytotoxic T cell
Th: T helper cell.
